# Base-Pair Opening Dynamics Study of Fluoride Riboswitch in the *Bacillus cereus CrcB* Gene

**DOI:** 10.3390/ijms22063234

**Published:** 2021-03-22

**Authors:** Juhyun Lee, Si-Eun Sung, Janghyun Lee, Jin Young Kang, Joon-Hwa Lee, Byong-Seok Choi

**Affiliations:** 1 Department of Chemistry, Korea Advanced Institute of Science and Technology (KAIST), 291 Daehak-ro, Yuseong-gu, Daejeon 34141, Korea; mkhm44@kaist.ac.kr (J.L.); skyn0507@kaist.ac.kr (S.-E.S.); janghlee86@kaist.ac.kr (J.L.); 2 Department of Chemistry and RINS, Gyeongsang National University, Gyeongnam 52828, Korea

**Keywords:** fluoride riboswitch, aptamer, long-range interaction, tertiary interaction, NMR, base-pair opening, hydrogen exchange dynamics

## Abstract

Riboswitches are segments of noncoding RNA that bind with metabolites, resulting in a change in gene expression. To understand the molecular mechanism of gene regulation in a fluoride riboswitch, a base-pair opening dynamics study was performed with and without ligands using the *Bacillus cereus* fluoride riboswitch. We demonstrate that the structural stability of the fluoride riboswitch is caused by two steps depending on ligands. Upon binding of a magnesium ion, significant changes in a conformation of the riboswitch occur, resulting in the greatest increase in their stability and changes in dynamics by a fluoride ion. Examining hydrogen exchange dynamics through NMR spectroscopy, we reveal that the stabilization of the U45·A37 base-pair due to the binding of the fluoride ion, by changing the dynamics while maintaining the structure, results in transcription regulation. Our results demonstrate that the opening dynamics and stabilities of a fluoride riboswitch in different ion states are essential for the genetic switching mechanism.

## 1. Introduction

A number of metabolite-binding riboswitches have been discovered in the noncoding RNA structures in bacterial genomes. Riboswitches bind to a variety of metabolites and recruit conformational changes that control the transcription, translation, or splicing [1,2,3]. In most instances, riboswitches are composed of two parts: an aptamer domain that specifically binds a metabolite, and an expression platform domain that operates a genetic switch [4,5]. The conserved ligand-binding domain is flexible and undergoes ligand-induced conformational transitions, which lead to changes in the folding of the expression platform [6]. To date, diverse types of ligands have been discovered—e.g., anions, coenzymes, metal ions, amino acids, purines, and their derivatives—which maintain intracellular homeostasis [7,8].

Among the diverse type of ligands, fluoride riboswitches are particularly affected by the concentration of fluoride ions. Fluoride ions are abundant in the environment and toxic to bacterial growth; therefore, the control of fluoride concentration in cells is important to the bacterial lifecycle and survival [9,10,11]. The *CrcB* motif is a conserved domain that resides in the 5′ untranslated regions (UTRs) of genes encoding DNA repair, ion transporters (K^+^, Cl^−^), and formate hydrogen lyase. The expression of *CrcB* is important for reducing the fluoride concentration in cells and its toxicity [10]. The highly conserved nucleotides of the *CrcB* motif greatly change their conformation in the presence of NaF, as observed using the in-line probing method [11]. The fluoride binding to aptamer domain leads to the formation of the anti-terminator stem that allows access of RNA polymerase for activation of transcription. In contrast, in absence of fluoride ion, the fluoride riboswitch forms the terminator stem for the termination of transcription [4].

The ligand-bound (holo) structure of *Thermotoga petrophila* and the ligand-free (apo) structure of *B. cereus* riboswitches were previously determined by X-ray crystallography and NMR experiments, respectively. Most of the riboswitches form tight binding pockets to encapsulate their ligands in their binding pockets. X-ray crystallography studies demonstrated how the negatively-charged RNA strongly binds to the negatively-charged fluoride ion and how RNA architecture adopts the fluoride ligand selectively against other halide ions. The binding pocket of the fluoride aptamer is located in the internal loop and the pocket is stabilized by the formation of pseudoknot and long-range interaction, including a reverse Watson–Crick base pair and a reverse Hoogsteen pair [12]. NMR experiments of the fluoride riboswitch revealed that the solution structure of the riboswitch in the presence of Mg^2+^ exists in a highly similar conformation to the holo state. Recently, the mechanism of the fluoride riboswitch has been characterized using chemical exchange saturation transfer (CEST) NMR spectroscopy [13,14,15,16], which identified an excited state of the ligand-free aptamer (apo-ES) that exists in low population with short lifetimes. In particular, the apo-ES of the U45 imino proton transiently unlocks the A37·U45 base pair (called a linchpin) and affects the transcription termination by forming a kinetically favorable U45·A65 pair. In contrast, ligand binding leads to a single stable conformation (holo state) that ensures the transcription activation by allosteric inhibition of the ES transition [15,16]. Despite the characterization of the slow chemical exchange in the fluoride riboswitch, the mechanism of the fluoride riboswitch remains unclear.

Here, we identified the base-pair dynamics of the fluoride riboswitch using an NMR hydrogen exchange experiment. The NMR hydrogen exchange experiments provided information about the thermodynamics and kinetics for base-pair opening on time scales slower than milliseconds. These NMR data can also be used to probe the structural and dynamic features of the base pairs required for their biological function. We also determined that the fluoride aptamer domain and the expression platform show differences in their conformational dynamics and stability in the presence of different ion states. This study revealed that the dynamic features induced by fluoride binding play an important role in the biological function of the fluoride riboswitch.

## 2. Results

### 2.1. Resonance Assignments of CrcB Motif in the Free, Apo, and Holo forms

**Figure 1:**
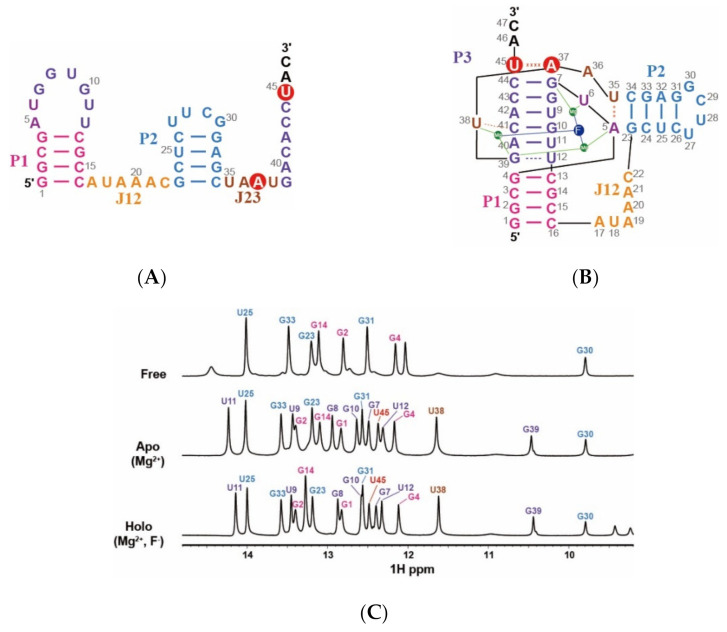
Secondary conformation changes in the *CrcB* motif according to ionic conditions. Each part of the *CrcB* aptamer is highlighted in different colors and shown next to it. The Watson–Crick base pairs are shown as solid lines and the tertiary interactions are shown as dashed lines. The A37·U45 base-pair is highlighted with a red circle. Fluoride and magnesium ions are labeled using blue and green circles, respectively. (**A**) Secondary structure and sequence of the *CrcB* motif in the free state (in the absence of Mg^2+^ and F^−^) and (**B**) in the apo state (in presence of Mg^2+^) and holo state (in the presence of Mg^2+^ and F^−^). (**C**) 1D imino proton spectra of free, apo, and holo states at 25 °C.

**Figure 2:**
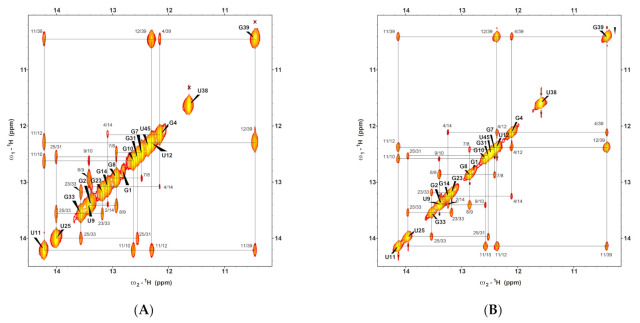
Imino proton resonance assignments of a fluoride riboswitch in the (**A**) apo and (**B**) holo states by Watergate NOESY spectra at 25 °C. Solid lines indicate imino–imino NOE connectivities.

The construct of *B. cereus CrcB* aptamer was derived from a modified RNA sequence reported by Zhao et al. (2014) to increase the stability and minimize spectral overlap. This sequence was designed so that the GGUU loop at the P2 stem was replaced with a UUCG loop, and A9·U42 was changed to U9·A42 [14,15]. The 1D imino proton spectra of the fluoride riboswitch at 25 °C (298 K) are shown in Figure 1C, and the assignments of each state were performed through the analysis of the NOESY spectra (Figure 2 and Figure S1).

From the results of a previous study, the fluoride riboswitch requires magnesium ions to recognize fluoride ions selectively, and remarkably undergoes Mg^2+^-induced structural changes [15]. Accordingly, in the presence of magnesium ions (apo), an additional P3 stem was formed in the *CrcB* aptamer, which proved to significantly increase the number of 1D amino proton peaks, but resonances from P1and P2 stems were observed only in the free state. Comparing the free and apo states, a chemical shift was observed in the imino protons of G2, G31, and G33 (Figure 1).

In the presence of a fluoride ion (holo), we observed that the 1D proton spectrum is similar to its apo state; however, some imino protons in the P1 and P3 stems undergo chemical shifts by fluoride binding (Figure 1B,C). In the holo state compared to the apo state, the G8 and G10 protons resonate ~0.1 ppm upfield chemical shifts, and G14 resonance has downfield chemical shifts from 13.095 to 13.271 ppm. In particular, upfield-shifted G7 and downfield-shifted U45 signals were observed and their peak positions changed with each other due to the ligand binding. These results indicated that fluoride binding affects the conformation of the aptamer domain, although the tertiary structure does not undergo significant changes.

### 2.2. Imino Proton Exchange Rate of CrcB Motif

**Figure 3:**
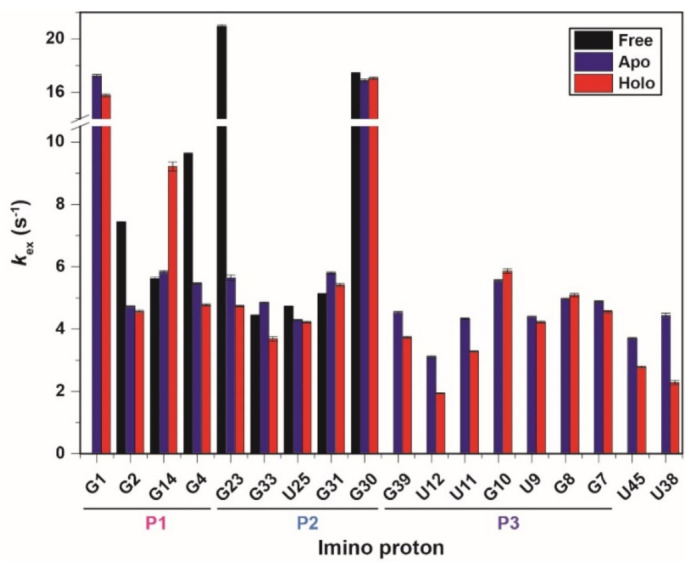
Exchange rate constants (*k*_ex_) of the *CrcB* aptamer at 25 °C determined by fitting to Equation (1), and the error bars indicate fitting errors: free (black), apo (blue), and holo (red) states. To identify the location of imino protons, the P1, P2, and P3 stems are shown under the residues.

The hydrogen exchange rates (*k*_ex_) of the fluoride riboswitch were confirmed at 25 °C (298 K) using water magnetization transfer experiments on the imino protons in all the states. Some imino protons showed significant differences in peak intensities due to a function of delay after water inversion (Figure S2). The *k*_ex_ values determined by fitting the curve using Equation (1) are shown in Figure 3 and Supplementary Table S1 [17,18]. For example, in the free state, rapidly exchanging imino protons (such as G23 and G30) show negative peaks at short delay times (50 ms), whereas the base pairs in the middle of the stem (such as G14, G33, U25, and G31) remain basically unchanged up to 100 ms (Figure S2A). Therefore, in the absence of ions, the imino protons located in the middle of the stem have significantly smaller *k*_ex_ values than the terminal base pair or loop (Figure 3).

In the presence of Mg^2+^ ions, the G2 and G4 imino protons in the P1 stem have smaller *k*_ex_ values of 4.73 and 5.45 s^−1^, respectively, compared with the *k*_ex_ values in the free state (G2: 7.47 s^−1^ and G4: 9.62 s^−1^, respectively). In particular, the G23 imino proton in the apo state has a 3.7-fold smaller *k*_ex_ value than in the free-state. In contrast, other imino protons in the P1 and P2 stem did not show significant differences in the *k*_ex_ values between the free and apo states. The base pair of imino protons produced by Mg^2+^ ions, such as the P3 stem, has *k*_ex_ values ranging from 3.1 to 5.56 s^−1^, similar to other base pairs. These results suggest that binding to the Mg^2+^ ion greatly stabilizes most of the aptamer domain, except for the residues of the P2 stem (Figure 3 and Supplementary Table S1).

In the holo state, except for G14, the imino protons in the P1 to P3 stems have similar or slightly smaller *k*_ex_ values to the apo state. The *k*_ex_ of the G14 imino proton is approximately 1.6-fold larger than in the ligand-free state. In contrast, the U12·G39 wobble base pair in the holo state has significantly smaller *k*_ex_ of 3.74 and 1.94 s^−1^ compared with *k*_ex_ values in the apo state (G39: 4.53 s^−1^ and U12: 3.10 s^−1^, respectively). The U38 and U45 residues, known as long-range interaction regions, have 1.9- and 1.3-fold smaller *k*_ex_ values than in the apo state, respectively (Figure 3 and Supplementary Table S1). Therefore, it is interpreted that the U38 and U45 increase the stability of the fluoride binding site. These results indicate that the residues of long-range interaction and the binding pocket slowly exchange protons in the presence of fluoride.

### 2.3. Base-Pair Opening Dynamics of CrcB Motif by the Tris Base Catalyst

The equilibrium constants for base-pair opening (*K*_op_) in the *CrcB* motif were determined by measuring the Tris-catalyzed imino proton exchange at 25 °C. Ammonia (NH_3_) is a much stronger catalyst than Tris and was used to determine *K*_op_ for the base pairs. NH_3_ was not used because the reaction of ammine complex formation was activated by the MgCl_2_ present in the buffer [19].

The base-pair opening is affected by the base catalyst, decreasing the peak intensities of imino protons. These results can be confirmed in the 1D imino proton spectra with the [Tris] difference in Figure S3. In all the spectra (free, apo, and holo), the base-pairs with stable interactions maintained their peak intensities even at higher concentrations of Tris base titration.

The inversion recovery experiments of the *CrcB* motif were performed to confirm the effect of [Tris] on the *K*_op_ of imino protons; the result for the imino protons is shown in Table 1. For most of the imino protons, the equilibrium constants for base-pair opening (*K*_op_) in the *CrcB* motif were determined from the slope of the linear correlation between *R*_1a_ and [B] using Equation (7). In some imino protons, the *K*_op_ and base-pair lifetimes (τ_0_ = 1/*k*_op_) in the *CrcB* motif were determined by curve fitting using Equation (6). These values were used to calculate the lifetime for base-pair opening (τ_open_ = 1/*k*_cl_) using the relation τ_open_ = τ_0_*K*_op_ (Figure S4 and Supplementary Table S2).

**Figure 4:**
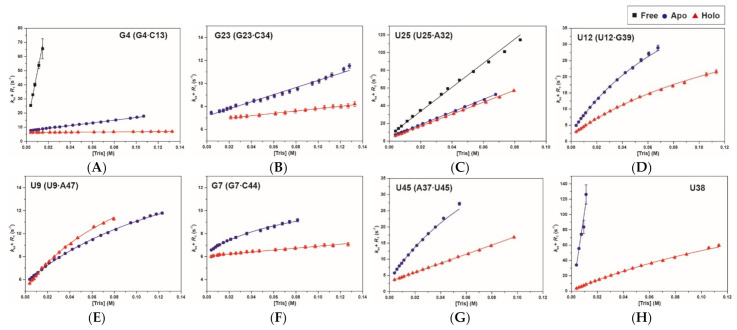
Tris-catalyzed hydrogen exchange data of major imino protons in the *CrcB* aptamer: (**A**) G4, (**B**) G23, (**C**) U25, (**D**) U12, (**E**) U9, (**F**) G7, (**G**) U45, and (**H**) U38 imino protons. The solid lines are the best fit to Equations (6) and (7), and the error bars represent the fitting errors during determination of *R*_1a_ (= *R*_1_ + *k*_ex_). [Tris] on the y-axis is the concentration of Tris (pH 8.0), which was used as the base catalyst. The *R*_1a_ values in the free, apo, and holo states are indicated by squares (black), circles (blue), and triangles (red), respectively.

**Figure 5:**
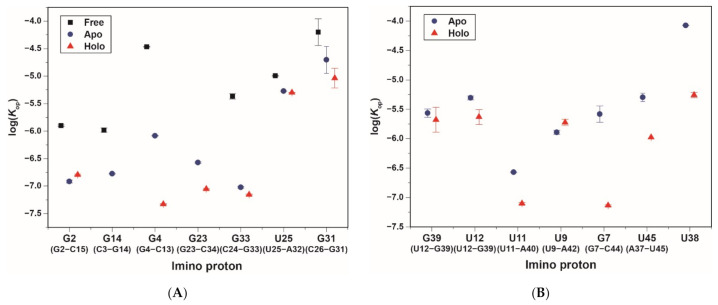
Equilibrium constants for base-pair opening (*K*_op_) of the *CrcB* aptamer determined by Tris-catalyzed NMR exchange experiments at 25 °C. (**A**) The imino protons of the P1 and P2 stems; (**B**) the imino protons in the P3 stem and imino protons with long-range interaction. The log(*K*_op_) values in the free, apo, and holo states are indicated by squares (black), circles (blue), and triangles (red), respectively.

In the presence of ions, the residues in the P1 and P2 stems, except for G4 and G31, have much smaller *K*_op_ values compared to the free state, whereas the *K*_op_ values are similar between the apo and holo states (Figure 4A and Figure 5A). The G2 and G4 imino protons of the P1 stem in the apo state have 10.5- and 6.1-fold smaller *K*_op_ values than in the free state. In the holo state, the *K*_op_ value of G2 is similar to the apo state (apo: 0.12 ± 0.007 × 10^−6^, holo: 0.16 ± 0.005 × 10^−6^), and G14 could not be determined because of the dependence of *R*_1a_ on Tris concentration (Table 1 and Figure S4B). In addition, the G4·C13 base pair in the holo state has 723- and 17-fold smaller *K*_op_ values than in the free and apo states, respectively. The G23 residue in the free state was not observed in imino proton resonance due to the presence of Tris (Figure S4A), while the *K*_op_ values in the other states were determined (apo: 0.27 ± 0.007 × 10^−6^ and holo: 0.089 ± 0.002 × 10^−6^; Figure 4B). In the presence of Mg^2+^, the *K*_op_ values of G33 and U25 are smaller than in the free state, but similar to the holo state (Figure 4C and Figure 5A). The G31 imino proton determined not only the *K*_op_ (free: 63 ± 35 × 10^−6^, apo: 20 ± 11 × 10^−6^; holo: 9.17 ± 4 × 10^−6^), but also base-pair lifetimes (free: 67 ± 0.4 ms; apo: 69 ± 0.8 ms; holo: 67 ± 1 ms) by curve fitting (Figure S4G).

In the presence of fluoride, the imino protons related to the binding pocket (G7, G39, and U38) have smaller *K*_op_ values compared with the apo state (Figure 5B). Therefore, the fluoride binding affects the base-pair stability of the binding pocket compared with other regions. In the fluoride-bound state, the G39 and U12 imino protons, which formed a wobble base pair, have 1.3- and 2.1-fold smaller *K*_op_ than the apo state, respectively. Interestingly, the U12 imino proton in the apo and holo states is less stable (larger *K*_op_) and more dynamic (shorter τ_0_ and τ_open_) compared with G39, while base-pairing with each other (Figure 4D and Table 1). The holo state of the G7 imino proton shows much less dependence on Tris concentration compared with the apo state, resulting in a 35-fold smaller *K*_op_ value (Figure 4F). Surprisingly, the U9 imino proton of the F^−^-bound aptamer has a 1.4-fold larger *K*_op_ value than in the apo state, although the τ_0_ values were almost the same (Figure 4E and Table 1). The *K*_op_ for the G8 and G10 imino protons, which showed small changes in *R*_1a_, could not be determined or have small *K*_op_ values (Figure S4K,M). In particular, the tertiary interaction regions (such as U45 and U38) have a significant effect on the base-pair stability through fluoride binding. Therefore, the U45 and U38 imino protons in the F^−^-bound state have 4.8- and 15-fold smaller *K*_op_, respectively, compared with the apo state (Figure 4 and Table 1). The resonance of U38 imino proton in the apo state disappears at a high concentration of Tris, but was confirmed in the holo state (Figure S3B,C).

These results indicated that the P1 and P2 stems are stabilized significantly by the binding of Mg^2+^ ions, but are less affected by a fluoride ion. In contrast, the imino protons of the P3 stem and imino protons with tertiary interaction are more stabilized by the binding of F^−^ ions.

## 3. Discussion

The hydrogen exchange experiment using NMR, which has a long history of identifying nucleic acid stability, allows the characterization of base-pair opening dynamics. [17,18]. In this study, we analyzed the base-pair opening dynamics of the fluoride riboswitch and identified its ligand-dependent stability. In the presence of Mg^2+^, the *CrcB* aptamer forms a pseudoknot helix, the so-called P3 stem, and long-range tertiary interactions, resulting in a significant conformational change and the greatest increase in stability. Therefore, the resonances of the pseudoknot P3 stem are observed in the 1D imino proton NMR spectrum (Figure 1). The imino protons of the P1 and P2 stems in the presence of Mg^2+^ have significantly smaller *k*_ex_ and *K*_op_ values compared with in free state. In particular, the base pair of G4·C13 and G23·C34, near the A5·U35 (long-range reverse WC base pair), are the most stabilized in the P1 and P2 stems by the Mg^2+^ ions (Figure 5A).

The secondary and tertiary structures of fluoride riboswitch are highly similar between the apo and holo states, but the aptamer domain has different chemical transitions, as reported previously [15,16]. We also provide direct NMR evidence that the fluoride riboswitch has some differences in conformational dynamics between the apo and holo states. First, in the fluoride-binding holo state, the P1 stem becomes slightly more stable than in the fluoride-free apo state. The C3·G14 and G4·C13 base pairs in the presence of F^−^ have ~17.4-fold smaller *K*_op_ values than in the apo state because the stability of the P3 stem, which is continuously stacked with the P1 stem, changes with fluoride binding [12,15]. Second, the G23·C34 base pair in the P2 stem is significantly stabilized with three-fold smaller *K*_op_ due to the additional binding of the apo-state riboswitch to the fluoride anion (Table 1 and Figure 5A), although other base pairs in the P2 stem showed little effects (Table 1). Third, in the presence of a ligand, the residues in the pseudoknot P3 stem increase in stability compared to in the apo state. The *K*_op_ values of the G39 and G7 imino protons, which are involved in the binding pocket, decrease with ligand binding (Table 1). In the holo state, the *K*_op_ value of the U12 base-paired with G39 reduces two-fold compared with the apo state, and becomes similar to the *K*_op_ value of G39. Since U12 is located outside the binding pocket, the U12 imino proton is less stable and more dynamic than G39 in both states. In contrast, ligand binding destabilizes the U9 imino proton, increasing the *K*_op_ value from 1.28 ± 0.1 × 10^−6^ to 1.88 ± 0.2 × 10^−6^. Fourth, the tertiary interactions, such as U45 and U38 imino protons, are significantly stable upon binding of fluoride. The U45 imino proton, which forms a reversed Hoogsteen AU pair with A37, stabilizes in the presence of F^−^, showing 1.3- and 4.8-fold reduced changes in *k*_ex_ and *K*_op_ values, respectively. Interestingly, the U38 (2′OH and N3H3) imino proton forms hydrogen bonds with A40 (N7) and C41 (O2P), making these hydrogen bonds unstable in the presence of external catalyst due to p*K*_a_ differences [15]. In contrast, the tertiary interactions of U38 are strongly stabilized by fluoride binding. These results suggest that the apo state, which is relatively more unstable compared to the holo state, leads to ground- and excited state (GS–ES) conformational exchange in the aptamer domain.

In previous studies, the apo-ES of the U45 imino proton transiently unlocked the A37·U45 base pair (called the linchpin) and affected transcription termination by forming a kinetically favorable U45·A65 pair. In contrast, ligand binding leads to a single stable conformation (holo state), which ensures transcription activation by allosteric inhibition of the ES transition [15,16]. We also found that the interaction between the *CrcB* aptamer and F^−^ also leads to stabilization of the U45·A37 base pair with 4.8-fold smaller *K*_op_ (Table 1). Therefore, due to the difference in stability of the U45 imino proton, the fluoride riboswitch regulates transcription, maintaining a highly similar structure between the ligand-free and -bound states.

Taken together, our data suggest that the functional switching of the fluoride riboswitch aptamer can be activated by a two-step pathway: (1) conformational change induced by magnesium cations; and (2) dynamic change induced by fluoride anions.

## 4. Materials and Methods

### 4.1. Sample Preparation

RNA samples were prepared by in vitro transcription from DNA templates, which was derived using the method of Zhao et al. (2014) using T7 polymerase [13], with some modifications. After transcription, RNA fragments were extracted by ethanol precipitation, and then purified by 250 mL large-scale denaturing polyacrylamide gel (1× TBE, 7M urea, 12% acrylamide). RNA was eluted from the polyacrylamide gels using the Elutrap system (Whatman, Maidstone, UK). The collected RNA samples were concentrated using Amicon (Millipore, MA, USA) and ethanol precipitation [20]. The pellet was dissolved in distilled water and exchanged to sample (free) buffer (10 mM sodium phosphate, pH 6.4, 50 mM potassium chloride, 50 μM EDTA) using Amicon. The Apo (Mg^2+^-bound) sample was exchanged by adding 2 mM MgCl_2_ under the same buffer condition as the free sample. The holo (F^−^-bound) sample was exchanged under the same buffer condition with an additional 2 mM MgCl_2_ and 10 mM NaF. All samples were quantified by UV measurement at 260 nm, and concentrated to ~1 mM. RNA samples under these conditions were used in the water magnetization transfer and NOESY experiments. Before measurement with NMR spectroscopy, 10% D_2_O was added to the RNA samples.

For the Tris-catalyzed experiment, the RNA samples were exchanged to Tris buffer (10 mM Tris-d_11_-HCl, pH 8.0, 50 mM KCl, 50 μM EDTA) using Amicon. Under the Tris buffer conditions, the apo sample was exchanged by adding 2 mM MgCl_2_, and the holo sample was exchanged by adding 2 mM MgCl_2_ and 10 mM NaF. The Tris-HCl concentration was increased from 3.91 to 132 mM by adding 0.5 M Tris-HCl stock solution (0.5 M Tris-d_11_ (pH 8.0 at 25 °C), 50 mM KCl, 50 μM EDTA). The pH of the sample, which was dissolved in Tris-HCl buffer, was calculated using the equation ∆p*K*_a_ = −0.031 × ∆*T*. The ∆*T* is the difference of temperature between two pH measureing pionts.

### 4.2. NMR Experiments

NMR experiments were conducted on Bruker 700 MHz and 800 MHz spectrometer (Korea Basic Science Institute, Ochang, Korea). All data were processed with NMRPIPE [21] and analyzed by Sparky [22]. The imino protons of *CrcB* motif were assigned using water suppression NOESY at 298 K. The longitudinal relaxation rate constants (*R*_1a_ = 1/*T*_1_) were determined by semi-selective inversion recovery *T*_1_ measurements on imino protons. The longitudinal relaxation rate constants of water (*R*_1w_) were determined by selective inversion recovery using the DANTE sequence. The hydrogen exchange rates (*k*_ex_) of the imino protons were measured using water magnetization transfer [17,18]. The intensities of the imino protons were measured for 15 delay times (0.002–0.1 s). The exchange rates were obtained by fitting to Equation (1)

(1) $$\frac{I_{\left(t\right)}}{I_{0}}=1-2\frac{k_{ex}}{\left(R_{1w}-R_{1a}\right)}\left(e^{-R_{1a}t}-e^{-R_{1w}t}\right)$$

where *I*_0_ and *I*_(*t*)_ are the peak intensities of the imino proton in the water magnetization transfer experiments at times zero and *t*, respectively.

### 4.3. Hydrogen Exchange Theory

The imino proton exchange, consisting of base-pair opening and proton transfer to a base catalyst, has been previously described, and will be briefly explained [17,18]. The proton transfer rate (*k_tr_*) from the nucleotide to the base catalyst is represented by Equation (2)

(2) $$k_{tr}=k_{i}\left[B\right]+k_{int}=\frac{k_{coll}}{1+10^{∆pK_{a}}}\left[B\right]+k_{int},$$

where *k_i_* is the rate constant of imino proton transfer induced by a base catalyst, *k_int_* is the exchange rate constant catalyzed by an intrinsic base, *k_coll_* is the collision rate constant, [B] is the concentration of the externally added base catalyst such as Tris, ∆*pK_a_* is the *pK_a_* difference between the nucleoside and the base. Therefore, the rate constant for imino proton exchange (*k_ex_*), which catalyzed by the added and internal base, is given by Equation (3)

(3) $$k_{ex}=\frac{k_{op}k_{tr}}{k_{cl}+k_{tr}},$$

where *k_op_* and *k_cl_* are the opening and closing base-pair rate, respectively. Substituting Equation (2) into Equation (3) yields the the final equation of the *k_ex_*

(4) kex=kopkiB+kintkcl+kiB+kint=kopB+kopkintkiB+kint+kopKopki,

where *K_op_* (= *k_op_*/*k_cl_*) is the equilibrium constant for base-pair opening. Additionally, if the closing base-pair rate (*k_cl_*) is much larger than *k_i_* [B], Equation (4) is simplified to

(5) $$k_{ex}=K_{op}k_{i}\left[B\right].$$

The exchange rate constants of the imino proton were determined by measurement of the apparent longitudinal relaxation rate constants (*R*_1*a*_) at various concentrations of Tris-HCl buffer. The *R*_1*a*_ value of the imino proton is the sum of the relaxation constant of exchangeable imino protons except for exchange (*R*_1_), which is not affected by the external catalyst, and the rate constant of imino proton exchange (*k_ex_*). Therefore, the *R_1a_* for an imino proton is represented as

(6) R1a=R1+kex= R1+kopB+kopkintkiB+kint+kopKopki,

and the *K_op_* and *k_op_* values for the base-pair opening dynamics can be determined by curve fitting *R*_1*a*_ as a function of the base catalyst concentration with Equation (6). In addition, under certain conditions where *k_i_* [B] is much larger than *k_int_*

(7) $$R_{1a}=R_{1}+k_{ex}=R_{1}+K_{op}k_{i}\left[B\right].$$

The *K_op_* value can be determined by linear fitting with Equation (7) between the *R*_1*a*_ and [B].

## 5. Conclusions

The base-pair opening dynamics of the fluoride riboswitch aptamer in *B. cereus* were analyzed using NMR in this study; we found that the stability of its secondary structure is specifically changed depending on the ligands. We provided direct NMR evidence of the conformational changes in the presence of Mg^2+^ and the dynamics changes with the addition of F^−^. The imino protons of the fluoride riboswitch are significantly stabilized by stepwise addition of Mg^2+^ and F^−^. C3·G14 and G4·C13 in the P1 stem and G23·C34 located in the P2 stem were found to be the most stable base pairs by analyzing the exchange experiments using Tris as a base catalyst. These studies also revealed that the residues in the pseudoknot P3 stem are stabilized in the presence of fluoride. The G39 and G7 imino protons involved in binding pocket have more stable interactions than in the ligand free-state. U45 and U38 imino protons related to the tertiary interactions are significantly stable upon binding of fluoride, which leads to the formation of a reversed Hoogsteen AU pair and hydrogen bonds, respectively. Based on our results, we suggest that the fluoride riboswitch regulates the gene expression through a two-step pathway that consists of conformational changes due to Mg^2+^ and dynamics changes due to F^−^. The results indicate that the dynamics change due to ligand binding is essential to the biological function of the fluoride riboswitch.

## Figures and Tables

**Table 1:** Base-pair dissociation constants (*K*_op_) and base-pair lifetimes (τ_0_ = 1/*k*_op_) of the *CrcB* motif determined by the Tris-catalyzed NMR exchange experiments at 25 °C ^a.^

Substructure	Base Pair	Imino Proton		Free	Apo	Holo
P1	G2·C15	G2	*K*_op_ (×10^−6^)	1.26 ± 0.04	0.12 ± 0.007	0.16 ± 0.005
			τ_0_ (ms)	n.d. ^b^	n.d. ^b^	n.d. ^b^
	C3·G14	G14	*K*_op_ (×10^−6^)	1.04 ± 0.09	0.17 ± 0.002	<0.01 × 10^−6^
			τ_0_ (ms)	n.d. ^b^	n.d. ^b^	n.d. ^b^
	G4·C13	G4	*K*_op_ (×10^−6^)	34 ± 0.4	0.82 ± 0.01	0.047 ± 0.001
			τ_0_ (ms)	n.d. ^b^	n.d. ^b^	n.d. ^b^
P2	G23·C34	G23	*K*_op_ (×10^−6^)	n.d.^d^	0.27 ± 0.007	0.089 ± 0.002
			τ_0_ (ms)		n.d. ^b^	n.d. ^b^
	C24·G33	G33	*K*_op_ (×10^−6^)	4.29 ± 0.5	0.095 ± 0.003	0.070 ± 0.002
			τ_0_ (ms)	52 ± 1	n.d. ^b^	n.d. ^b^
	U25·A32	U25	*K*_op_ (×10^−6^)	10 ± 0.1	5.32 ± 0.03	5.03 ± 0.03
			τ_0_ (ms)	n.d. ^b^	n.d. ^b^	n.d. ^b^
	C26·G31	G31	*K*_op_ (×10^−6^)	63 ± 35	20 ± 11	9.17 ± 4
			τ_0_ (ms)	67 ± 0.4	69 ± 0.8	67 ± 1
P3	U12·G39	G39	*K*_op_ (×10^−6^)	n.d. ^e^	2.72 ± 0.4	2.10 ± 1
			τ_0_ (ms)		70 ± 2	104 ± 6
	U12·G39	U12	*K*_op_ (×10^−6^)	n.d. ^e^	4.93 ± 0.4	2.34 ± 0.7
			τ_0_ (ms)		15 ± 0.6	20 ± 4
	U11·A40	U11	*K*_op_ (×10^−6^)	n.d. ^e^	0.27 ± 0.004	0.079 ± 0.002
			τ_0_ (ms)		n.d. ^b^	n.d. ^b^
	G10·C41	G10	*K*_op_ (×10^−6^)	n.d. ^e^	0.12 ± 0.003	<0.01 × 10^−6 c^
			τ_0_ (ms)		n.d. ^b^	n.d. ^b^
	U9·A42	U9	*K*_op_ (×10^−6^)	n.d. ^e^	1.28 ± 0.1	1.88 ± 0.2
			τ_0_ (ms)		51 ± 1	51 ± 2
	G8·C43	G8	*K*_op_ (×10^−6^)	n.d. ^e^	<0.01 × 10^−6^	<0.01 × 10^−6^
			τ_0_ (ms)		n.d. ^b^	n.d. ^b^
	G7·C44	G7	*K*_op_ (×10^−6^)	n.d. ^e^	2.61 ± 0.8	0.073 ± 0.002
			τ_0_ (ms)		84 ± 3	n.d. ^b^
Long-range interactions	A37·U45	U45	*K*_op_ (×10^−6^)	n.d. ^e^	5.05 ± 0.8	1.05 ± 0.007
			τ_0_ (ms)		15 ± 1	n.d. ^b^
	U38·C41 and A40	U38	*K*_op_ (×10^−6^)	n.d. ^e^	84 ± 0.8	5.52 ± 0.6
			τ_0_ (ms)		n.d. ^b^	5.61 ± 0.4

^a^ Parameters used in the calculation: *k*_coll_ = 1.5 × 10^9^ s^−1^, p*K*_a_ (G−NH1) = 9.24, p*K*_a_ (U−NH3) = 9.20, p*K*a (Tris, 25 °C) = 8.192; sample conditions: 10 mM Tris (pH 8.0 at 25 °C), 50 mM KCl, 50 μM EDTA (pH 8.0) (for free state)/adding 2 mM MgCl_2_ (for apo state)/2 mM MgCl_2_, 10 mM NaF (for holo state). [Tris] total = 10–339 mM, 25 °C. The errors for these values were determined from the curve fitting using Equation (6) and linear fitting using Equation (7). ^b^ Not determined. ^c^ These resonances are partially overlapped with another resonance and this overlap may lead to a systematic error in *K*_op_. ^d^ Not available because the imino proton resonance disappeared. ^e^ No imino proton resonance.

## Data Availability

The data presented in this study are available in insert article or Supplementary Material here.

## Supplementary Materials

The following are available online at https://www.mdpi.com/1422-0067/22/6/3234/s1, Figure S1: Imino proton resonance assignments of a fluoride riboswitch in the free state by Watergate NOESY spectra at 25 °C.; Figure S2: 1D 1H spectra of water magnetization transfer experiments showing imino protons of the fluoride riboswitch in the (A) free, (B) apo, and (C) holo states; Figure S3: 1D spectra of the *CrcB* aptamer measured with increasing Tris concentrations in a 90% H_2_O/10% D_2_O solution containing 10 mM Tris (pH 8.0 at 25 °C), 50 mM KCl, 50 μM EDTA (pH 8.0); Figure S4: Hydrogen exchange data of the *CrcB* aptamer. The *R*_1_*_a_* of the imino protons as a function of the Tris concentration in NMR buffer at 25 °C; Table S1: Hydrogen exchange rate constants (*k*_ex_, s^−1^) of the *CrcB* aptamer; Table S2: Base-pair dissociation constants (*K*_op_), base-pair lifetimes (τ_0_ = 1/*k_op_*), and lifetimes for base-pair opening (τ_open_ = 1/*k_cl_*) of the *CrcB* motif determined by the Tris-catalyzed NMR exchange experiments at 25 °C.
