# Supplementary material for: Base-Pair Opening Dynamics Study of Fluoride Riboswitch in the Bacillus cereus CrcB Gene
Source: Int J Mol Sci. 2021 Mar 22;22(6):3234. doi: 10.3390/ijms22063234 (PMC8004769; doi:10.3390/ijms22063234)
Supplement: Supplementary file 1 [file ijms-22-03234-s001.pdf]

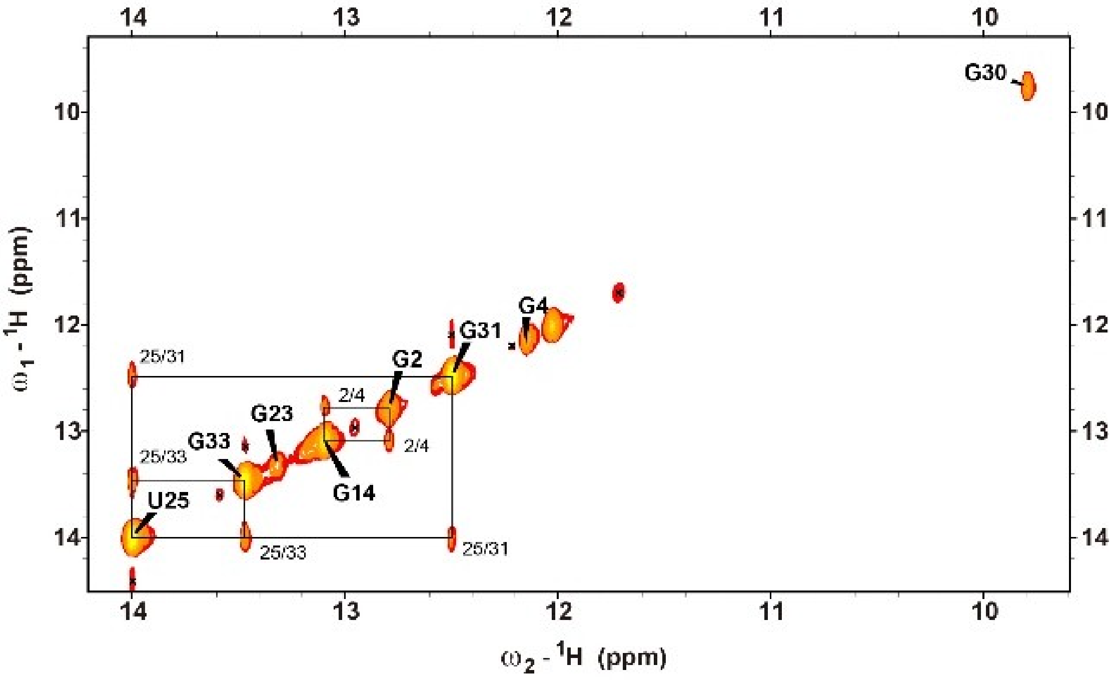

Figure S1. Imino proton resonance assignments of fluoride riboswitch in the free state by Watergate NOESY spectra at 25 °C.

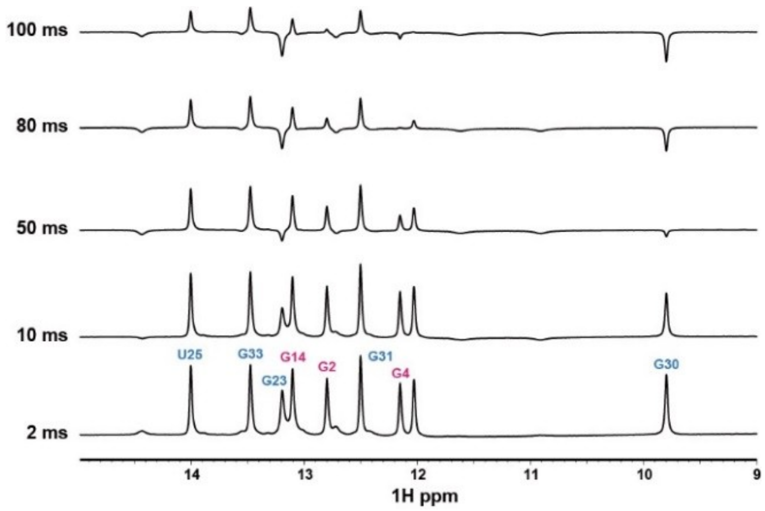

(**A**)

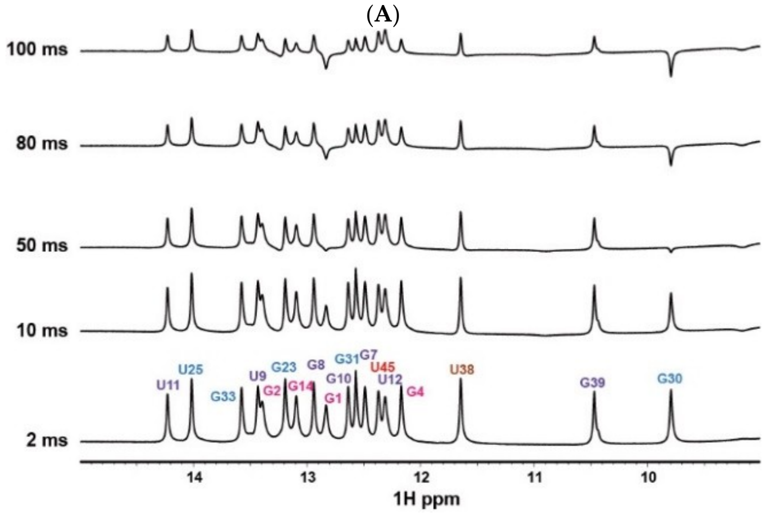

(**B**)

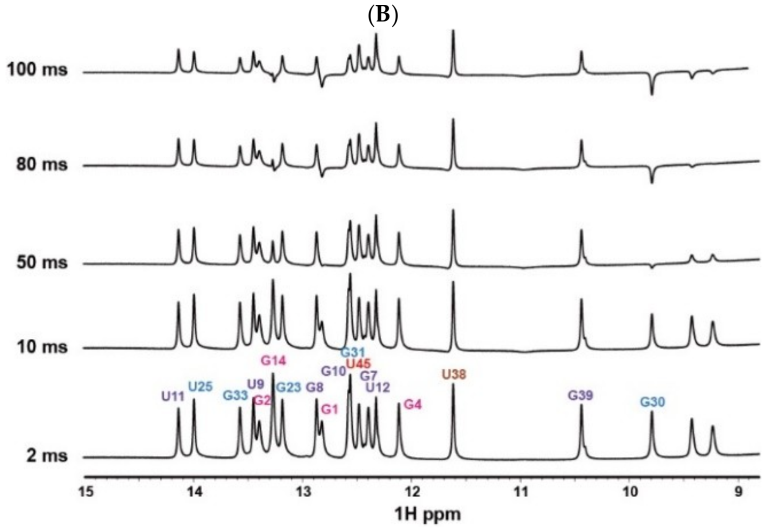

(**C**)

Figure S2. 1D ^{1}H spectra of water magnetization transfer experiments showing imino protons of the fluoride riboswitch in the (**A**) free, (**B**) apo and (**C**) holo states. The control spectrum with no selective water inversion is indicated at the bottom. The delay times between the selective water inversion and acquisition pulses are shown on the left side of the spectra.

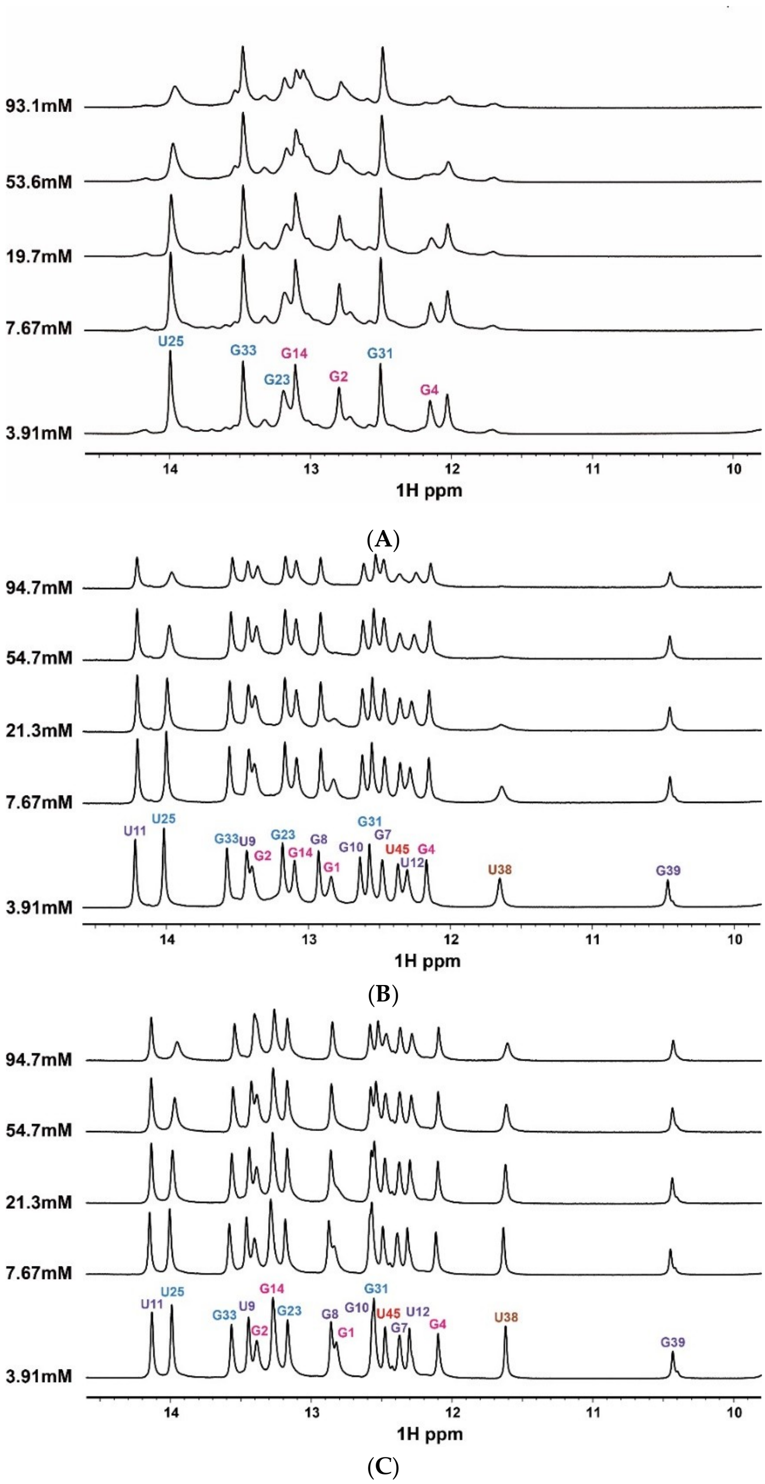

Figure S3. 1D spectra of *CrcB* aptamer measured with increasing Tris concentration in a 90% H_2O/10% D_2O solution containing 10mM Tris (pH 8.0 at 25 °C), 50mM KCl, 50uM EDTA (pH 8.0). The Tris concentrations are shown to the left of each spectrum. (**A**) free, (**B**) apo(add 2mM $MgCl_2$) and (**C**) holo(add 2mM $MgCl_2$ and 10mM NaF).

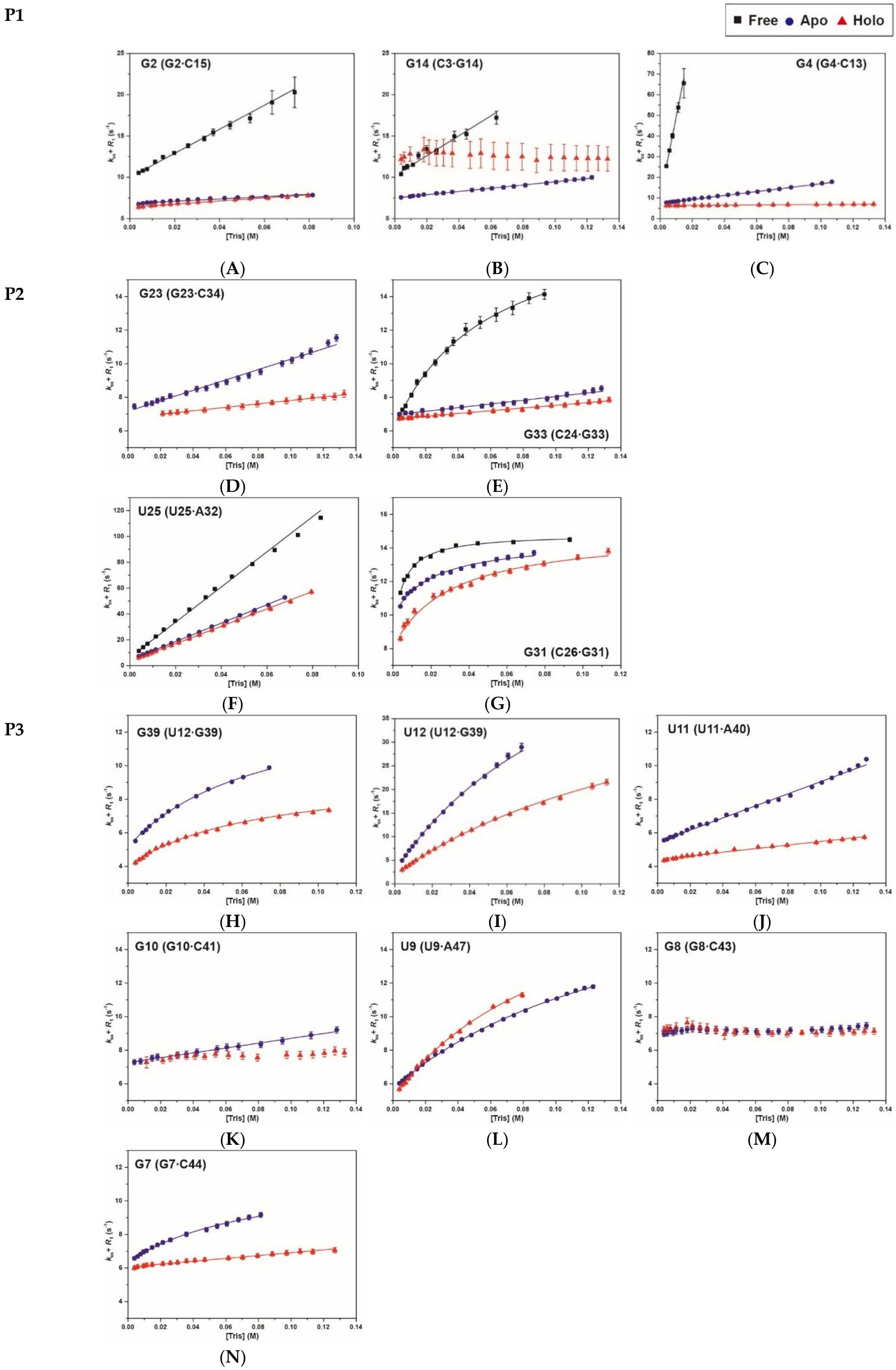
P1
Free
Apo
Holo
G2 (G2·C15)
G14 (C3·G14)
G4 (G4·C13)
(A)
(B)
(C)
P2
G23 (G23·C34)
G33 (C24·G33)
(D)
(E)
U25 (U25·A32)
G31 (C26·G31)
(F)
(G)
P3
G39 (U12·G39)
U12 (U12·G39)
U11 (U11·A40)
(H)
(I)
(J)
G10 (G10·C41)
U9 (U9·A47)
G8 (G8·C43)
(K)
(L)
(M)
G7 (G7·C44)
(N)
[Tris] (M)
$k_{ex} + R_1$ (s^{-1})

Loong-range interaction

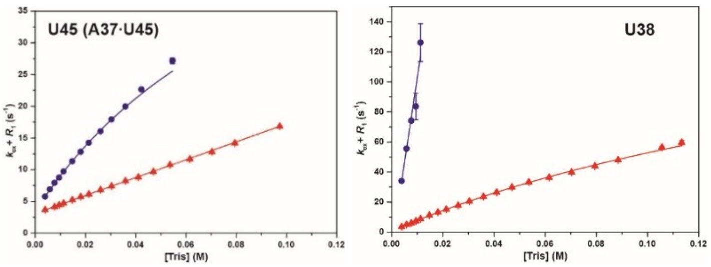

(**O**) (**P**)

Figure S4. Hydrogen exchange data of the *CrcB* aptamer. The R_{1a} of the imino protons as a function of the Tris concentration in NMR buffer at 25 °C. The location of the imino protons in the *CrcB* aptamer is marked on the left.

Supplementary table 1. Hydrogen exchange rate constants (k_{ex}, s^{-1}) of the *CrcB* aptamer.

**Base pair**	**Imino proton**	**Free**	**Apo**	**Holo**
G1·C16	G1	- [a]	17.3 ± 0.09	15.8 ± 0.1
G2·C15	G2	7.47 ± 0.02	4.73 ± 0.02	4.56 ± 0.04
C3·G14	G14	5.63 ± 0.03	5.83 ± 0.05	9.22 ± 0.1
G4·C13	G4	9.62 ± 0.03	5.45 ± 0.03	4.77 ± 0.04
G23·C34	G23	20.9 ± 0.1	5.65 ± 0.09	4.74 ± 0.03
C24·G33	G33	4.44 ± 0.01	4.85 ± 0.03	3.68 ± 0.08
U25·A32	U25	4.72 ± 0.02	4.29 ± 0.02	4.22 ± 0.02
C26·G31	G31	5.12 ± 0.02	5.80 ± 0.03	5.42 ± 0.04
U27·G30	G30	17.4 ± 0.04	16.9 ± 0.1	17.0 ± 0.09
U12·G39	G39	- [a]	4.53 ± 0.03	3.74 ± 0.04
U12·G39	U12	- [a]	3.10 ± 0.04	1.94 ± 0.02
U11·A40	U11	- [a]	4.33 ± 0.02	3.28 ± 0.02
G10·C41	G10	- [a]	5.56 ± 0.03	5.86 ± 0.07
U9·A42	U9	- [a]	4.39 ± 0.03	4.22 ± 0.03
G8·C43	G8	- [a]	4.97 ± 0.03	5.09 ± 0.05
G7·C44	G7	- [a]	4.88 ± 0.03	4.56 ± 0.04
A37·U45	U45	- [a]	3.70 ± 0.03	2.79 ± 0.03
	U38	- [a]	4.44 ± 0.07	2.28 ± 0.07

[a] No imino proton resonance.

Supplementary table 2. Base-pair dissociation constants (K_{op}), base-pair lifetimes ($\tau_0 = 1/k_{op}$) and lifetimes for base-pair opening ($\tau_{open} = 1/k_{cl}$) of the *CrcB* motif determined by the Tris-catalyzed NMR exchange experiments at 25 °C[a].

Substructure	Base pair	Imino proton		Free	Apo	Holo
P1	G2·C15	G2	K_{op} (x10^{-6})	1.26 ± 0.04	0.12 ± 0.007	0.16 ± 0.005
			τ_0 (ms)	n.d.[b]	n.d.[b]	n.d.[b]
			τ_{op} (ns)			
			k_{int} (x10^6)			
	C3·G14	G14	K_{op} (x10^{-6})	1.04 ± 0.09	0.17 ± 0.002	< 0.01 x 10^{-6}
			τ_0 (ms)	n.d.[b]	n.d.[b]	n.d.[b]
			τ_{op} (ns)			
			k_{int} (x10^6)			
	G4·C13	G4	K_{op} (x10^{-6})	34 ± 0.4	0.82 ± 0.01	0.047 ± 0.001
			τ_0 (ms)	n.d.[b]	n.d.[b]	n.d.[b]
			τ_{op} (ns)			
			k_{int} (x10^6)			
P2	G23·C34	G23	K_{op} (x10^{-6})	n.d.[d]	0.27 ± 0.007	0.089 ± 0.002
			τ_0 (ms)		n.d.[b]	n.d.[b]
			τ_{op} (ns)			
			k_{int} (x10^6)			
	C24·G33	G33	K_{op} (x10^{-6})	4.29 ± 0.5	0.095 ± 0.003	0.070 ± 0.002
			τ_0 (ms)	52 ± 1	n.d.[b]	n.d.[b]
			τ_{op} (ns)	224 ± 27		
			k_{int} (x10^6)	2.03 ± 0.2		
	U25·A32	U25	K_{op} (x10^{-6})	10 ± 0.1	5.32 ± 0.03	5.03 ± 0.03
			τ_0 (ms)	n.d.[b]	n.d.[b]	n.d.[b]
			τ_{op} (ns)			
			k_{int} (x10^6)			
	C26·G31	G31	K_{op} (x10^{-6})	63 ± 35	20 ± 11	9.17 ± 4
			τ_0 (ms)	67 ± 0.4	69 ± 0.8	67 ± 1
			τ_{op} (ns)	4243 ± 2388	1359 ± 762	611 ± 253
			k_{int} (x10^6)	0.31 ± 0.1	1.57 ± 0.2	190 ± 0.3
P3	U12·G39	G39	K_{op} (x10^{-6})	n.d.[e]	2.72 ± 0.4	2.10 ± 1
			τ_0 (ms)		70 ± 2	104 ± 6
			τ_{op} (ns)		192 ± 32	219 ± 107
			k_{int} (x10^6)		2.98 ± 0.3	3.27 ± 1
	U12·G39	U12	K_{op} (x10^{-6})	n.d.[e]	4.93 ± 0.4	2.34 ± 0.7
			τ_0 (ms)		15 ± 0.6	20 ± 4
			τ_{op} (ns)		72 ± 6	47 ± 16
			k_{int} (x10^6)		0.55 ± 0.03	0.83 ± 0.2
	U11·A40	U11	K_{op} (x10^{-6})	n.d.[e]	0.27 ± 0.004	0.079 ± 0.002
			τ_0 (ms)		n.d.[b]	n.d.[b]
			τ_{op} (ns)			
			k_{int} (x10^6)			

	G10·C41	G10	K_{op} (x10^{-6})	n.d.[e]	0.12 ± 0.003	< 0.01 x 10^{-6} [c]
			τ_0 (ms)		n.d.[b]	n.d.[b]
			τ_{op} (ns)			
			k_{int} (x10^{6})			
	U9·A42	U9	K_{op} (x10^{-6})	n.d.[e]	1.28 ± 0.1	1.88 ± 0.2
			τ_0 (ms)		51 ± 1	51 ± 2
			τ_{op} (ns)		65 ± 5	96 ± 12
			k_{int} (x10^{6})		6.25 ± 0.3	3.69 ± 0.3
	G8·C43	G8	K_{op} (x10^{-6})	n.d.[e]	< 0.01 x 10^{-6}	< 0.01 x 10^{-6}
			τ_0 (ms)		n.d.[b]	n.d.[b]
			τ_{op} (ns)			
			k_{int} (x10^{6})			
	G7·C44	G7	K_{op} (x10^{-6})	n.d.[e]	2.61 ± 0.8	0.073 ± 0.002
			τ_0 (ms)		84 ± 3	n.d.[b]
			τ_{op} (ns)		219 ± 69	
			k_{int} (x10^{6})		5.20 ± 0.7	
Tertiary interactions	A37·U45	U45	K_{op} (x10^{-6})	n.d.[e]	5.05 ± 0.8	1.05 ± 0.007
			τ_0 (ms)		15 ± 1	n.d.[b]
			τ_{op} (ns)		73 ± 14	
			k_{int} (x10^{6})		0.72 ± 0.1	
	U38·C41 and A40	U38	K_{op} (x10^{-6})	n.d.[e]	84 ± 0.8	5.52 ± 0.6
			τ_0 (ms)		n.d.[b]	5.61 ± 0.4
			τ_{op} (ns)			31 ± 4
			k_{int} (x10^{6})			0.091 ± 0.02

[a] Parameters used in the calculation: k_{coll} = 1.5 × 10^{9} s^{-1}, pK_a (G–NH1) = 9.24, pK_a (U–NH3) = 9.20, pKa (Tris, 25 °C) = 8.192; sample conditions: 10 mM Tris (pH 8.0 at 25 °C), 50 mM KCl, 50 μM EDTA (pH 8.0) (for free state)/adding 2 mM $MgCl_2$ (for apo state)/2 mM $MgCl_2$, 10 mM NaF (for holo state). [Tris] total = 10–339 mM, 25 °C. The errors for these values were determined from the curve fitting using Equation (6) and linear fitting using Equation (7). [b] Not determined. [c] These resonances are partially overlapped with another resonance and this overlap may lead to a systematic error in K_{op}. [d] Not available because the imino proton resonance disappeared. [e] No imino proton resonance.
